# Structural evolution after oxidative pretreatment and CO oxidation of Au nanoclusters with different ligand shell composition: a view on the Au core†

**DOI:** 10.1039/d2cp04498f

**Published:** 2023-01-05

**Authors:** Vera Truttmann, Florian Schrenk, Carlo Marini, Mireia Palma, Maricruz Sanchez-Sanchez, Christoph Rameshan, Giovanni Agostini, Noelia Barrabés

**Affiliations:** a Institute of Materials Chemistry, TU Wien Getreidemarkt 9/165 Vienna 1060 Austria noelia.rabanal@tuwien.ac.at; b Chair of Physical Chemistry, Montanuniversität Leoben Franz-Josef-Straße 18 Leoben 8700 Austria; c ALBA Synchrotron Light Facility, Carrer de la Llum 2-26 Cerdanyola del Valles Barcelona 08290 Spain; d Institute of Chemical, Environmental and Bioscience Engineering, TU Wien Getreidemarkt 9/166 Vienna 1060 Austria

## Abstract

The reactivity of supported monolayer protected Au nanoclusters is directly affected by their structural dynamics under pretreatment and reaction conditions. The effect of different types of ligands of Au clusters supported on CeO_2_ on their core structure evolution, under oxidative pretreatment and CO oxidation reaction, was investigated. X-ray absorption and X-ray photoelectron spectroscopy studies revealed that the clusters evolve to a similar core structure above 250 °C in all the cases, indicating the active role of the ligand–support interaction in the reaction.

## Abstract 2

The structural dynamics of supported monolayer protected Au nanoclusters after pretreatment and catalytic CO oxidation have been probed by XAS and XPS.

## Graphical abstract

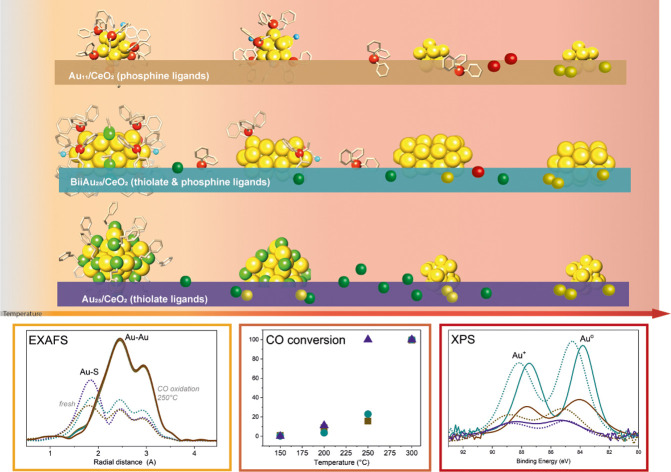

One of the biggest challenges in catalysis is to understand structure–activity relationships, which are required for a more targeted design of future catalysts.^1–3^ In that regard, gold nanoclusters are optimal model catalysts, since they can initially be prepared monodisperse, thus reducing the system complexity, and are quite active for several types of reactions,^1–3^ including CO oxidation.^4–18^ For this reaction especially, many different aspects have already been studied, including the dependence on cluster size/structure,^11,12,18^ dopant atoms,^4,6,15^ pretreatment,^4,5,8,9,11,13,14,16^ support,^8,10,13^ water content^13,14,17^ and nature of ligands.^7,9,11^ In our previous work, we investigated how different ligand environments influence the clusters’ catalytic behaviour. Indeed, we found striking differences between a set of [Au_25_(SC_2_H_4_Ph)_18_]TOA/CeO_2_ (“Au_25_/CeO_2_”) clusters in comparison to [Au_25_(PPh_3_)_10_(SC_2_H_4_Ph)_5_Cl_2_]Cl_2_/CeO_2_ (“Biico Au_25_/CeO_2_”) and Au_11_(PPh_3_)_7_Cl_3_/CeO_2_ (“Au_11_/CeO_2_”) clusters and their activity in CO oxidation: while the cluster only protected by thiolates reached 100% conversion when pretreated and reacted above 200 °C, the two clusters (partially) protected by phosphines required pretreatment temperatures of 300 °C to reach similar conversions. *In situ* transmission IR studies suggested that this might be related to the formation of ligand residues on the cluster–support interface, which hindered the catalytic reaction. Furthermore, (scanning) transmission electron microscopy ((S)TEM) images indicated cluster growth, but this was not considered to be the main cause of the activity differences.^7^ However, a thorough investigation focused on the evolution of the Au core was not conducted at this time. Nevertheless, previous investigations have shown that the removal of ligands often directly causes changes to the metallic core.^5,9,19–21^ Thus, both aspects can affect the catalytic activity and should therefore also be considered in structure–activity relationship investigations.

Our previous studies of the Au_38_(SC_2_H_4_Ph)_24_/CeO_2_ system by extended X-ray absorption fine structure (EXAFS) measurements have shown that the process of ligand removal leads to more drastic changes than originally expected: the ligands not only migrated onto the support, but also caused a partial migration of Au atoms away from the cluster core. This completely exposed the Au surface and interface at 250 °C, resulting in significantly increased activity in CO oxidation.^5^ Jin and coworkers also intensively investigated how the thiolate desorption influences the CO oxidation activity of Au_25_(SR)_18_/CeO_2_ catalysts and found that different gold species are formed upon ligand removal, of which the Au^*δ*+^ (*δ*: between 0 and 1) sites were found to be the major active sites in the catalytic reaction.^9^ Our previous study also noticed formation of both Au^+^ and Au^*δ*+^ sites through the oxidative pretreatment at 250 °C, the former of which mostly converted toward Au^*δ*+^ sites in the subsequent CO oxidation.^7^ For a series of CeO_2_ supported phosphine-protected clusters, the core structures appeared stable after thermal treatment at 120 °C while the ligands were shown to desorb from the clusters and interact with the support instead.^12^

A similar migration effect was assumed to take place in our previous study as well, however, a definitive proof by probing the state of the Au core was missing. Therefore, in addition to the previous kinetic tests and infrared studies,^7^ X-ray absorption spectroscopy (XAS) and X-ray photoelectron spectroscopy (XPS) measurements were performed with the same set of Au_11_/CeO_2_, Biico Au_25_/CeO_2_ and Au_25_/CeO_2_ catalysts. EXAFS at Au L_3_-edge indeed showed that the contribution of the ligands is negligible after 200 °C while they could still be detected in the catalyst system at this temperature previously,^7^ which confirms that they relocate to the support surface instead. Moreover, it appears that the difference in reactivity at 250 °C might be attributed to the ligand and its interaction with the support, since the final state of the Au cluster core after 250 °C is similar for the three samples. However, after 300 °C, a redispersion of both Au_11_/CeO_2_ and Biico Au_25_/CeO_2_ toward slightly smaller species was observed, which correlates with increased CO conversion.

## Experimental

The cluster catalysts used in this study were prepared and studied in previous work.^7^ The different species, [Au_25_(SC_2_H_4_Ph)_18_]TOA/CeO_2_ (“Au_25_/CeO_2_”), biicosahedral [Au_25_(PPh_3_)_10_(SC_2_H_4_Ph)_5_Cl_2_]Cl_2_/CeO_2_ (“Biico Au_25_/CeO_2_”) and Au_11_(PPh_3_)_7_Cl_3_/CeO_2_ (“Au_11_/CeO_2_”), were selected due to being common examples of Au nanoclusters possessing different ligand shell compositions (fully thiolate-protected, thiolate and phosphine-protected and fully phosphine-protected). A detailed description of the exact synthesis protocols, as well as on the catalytic CO oxidation measurements of the Au nanoclusters can be found elsewhere.^7^ The catalysts were pretreated under oxidative atmosphere (5% O_2_ in Ar) before CO oxidation (mixture of 1% CO and 2% O_2_ in Ar). Four different pretreatment and reaction maximum temperatures were tested for each catalyst: 150 °C, 200 °C, 250 °C and 300 °C.

Extended X-ray absorption fine structure measurements (EXAFS) at Au L_3_-edge were obtained at the NOTOS beamline of ALBA synchrotron (Spain). The supported cluster samples were grinded and pressed into pellets, which were mounted on a sample holder using Kapton tape. The unsupported clusters were dissolved in dichloromethane and dropcasted on Kapton tape for measuring. Consecutively, the samples were measured at room temperature in fluorescence mode by a Silicon Drift Detector. Data treatment and fitting was performed with the Artemis software package.^22,23^ The extracted EXAFS signal after background correction at pre- and post-edge was subsequently *k*^2^ weighted. A model with two shells (with Au–P/S and Au–Au) was used to fit the data, based on the resolved crystal structures of the clusters.^24–27^ Due to the similar scattering paths of Au–P and Au–S,^20^ those could not be resolved for Biico Au_25_/CeO_2_ and thus only a single path (Au–S) was used for fitting. One Debye Waller factor per different path (Au–Au, Au–P and Au–S) was used. Both an energy correction to the theoretical energy and an amplitude correction were applied. See Table S1 in the ESI† for details. Note that the path contributions are only above the noise level estimated as the intensity of the Fourier transformed EXAFS spectra at *R* = 10 Å if CN ≥ 0.3 (see also exemplary illustration in Fig. S5, ESI†).

X-Ray photoelectron spectroscopy (XPS) measurements of the clusters as synthesized, after oxidative pretreatment at 250 °C and after CO oxidation were done on a *in situ* near ambient pressure (NAP)-XPS system, equipped with a Phoibos 150 NAP hemispherical analyser and a XR 50 MF X-ray source (microfocus), all SPECS GmbH. Monochromatic Al K_α_ radiation was used to acquire the data, which was analysed with the CasaXPS software. Peaks were fitted after linear background subtraction with Gauss–Lorentz sum functions. The spectra were referenced to the Fermi edge and the C 1s signal (284.5 eV). Peak positions and full width at half-maximum (FWHM) were left unconstrained. Due to differential charging, the following samples were measured in 1 mbar N_2_ atmosphere instead of UHV conditions: Au_11_ as prepared, Au_25_ after reaction and Biico Au_25_ after oxidation.

## Results and discussion

**Fig. 1:**
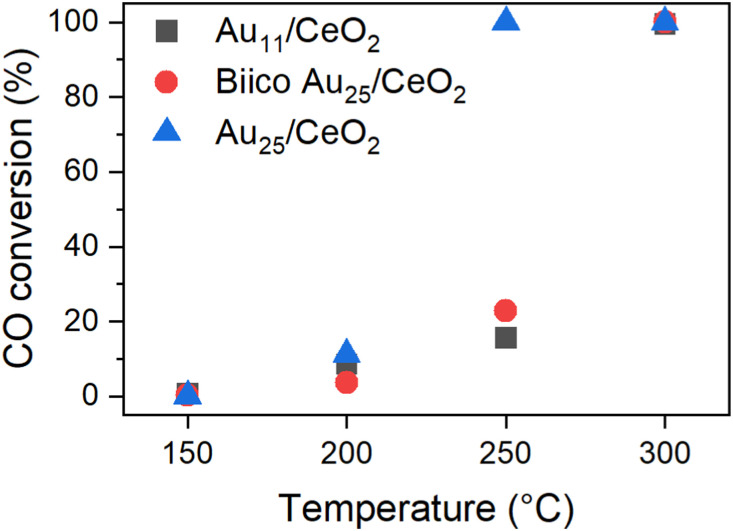
Catalytic activity of the Au nanocluster catalysts pretreated and reacted at the respective temperature. Normalized to 0.3 wt% Au and 15 mg catalyst. See ref. 7

The effects of different pretreatments and reaction temperatures were examined for a set of Au nanocluster catalyst in CO oxidation, as described and discussed in our previous publication.^7^ The most striking differences were observed for those samples pretreated and after reaction at 250 °C. Whereas the Au_25_/CeO_2_ catalyst showed a sudden increase in activity when pretreated at 250 °C, only about ≈20% conversion was achieved at that temperature with Au_11_/CeO_2_ and Biico Au_25_/CeO_2_. After pretreatment at 300 °C, no such differences were observed and all clusters exhibited high activity. Fig. 1 gives an overview of the conversion of the individual catalysts at the respective maximum temperatures; a more detailed discussion can be found elsewhere.^7^

This phenomenon was assumed to be related to the respective ligand environments: while both Au_11_/CeO_2_ and Biico Au_25_/CeO_2_ were partially protected by phosphine ligands, Au_25_/CeO_2_ is a purely thiolate-protected cluster. Indeed, *in situ* transmission IR studies and temperature programmed oxidation (TPO) measurements further affirmed the assumption that ligand residues were responsible for the different catalytic behaviour since they seemed to block the active sites. However, besides probing the particle size by (S)TEM and detecting an increase in size for all cluster catalysts, no further investigations of the dynamics of the Au core were carried out.^7^ Thus, to confirm that the dominant factor for the activity differences noticed was indeed the different original ligand shell, further characterization of the catalysts with focus on the state of the Au core were carried out by XAS and XPS.

**Fig. 2:**
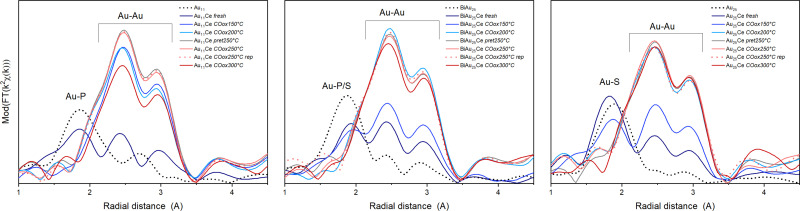
EXAFS data of the catalysts in *R*-space of the three catalysts at different stages, from left to right: Au_11_/CeO_2_, Biico Au_25_/CeO_2_ and Au_25_/CeO_2_. Each graph shows the spectrum of the unsupported clusters (black dotted), the supported catalysts (fresh; dark blue), the clusters after pretreatment and reaction at different temperatures (COoxXXX °C; blue or red solid lines), the catalyst after pretreatment at 250 °C but before reaction (pret250 °C; grey) and after 3 consecutive CO oxidation runs (COox250 °C rep; red dashed).

Fig. 2 shows the Fourier transform of the EXAFS spectra (*R*-space) and Table 1 the results of the EXFAS fitting of different states of all three cluster catalysts (unsupported cluster; fresh catalyst; after pretreatment and CO oxidation at 150 °C, 200 °C, 250 °C and 300 °C; only after oxidative pretreatment at 250 °C; after three consecutive CO oxidation experiments at 250 °C).

**Table 1:** Results of the EXFAS fitting results for the cluster catalysts at different stages

	Au_11_/CeO_2_				Biico Au_25_/CeO_2_				Au_25_/CeO_2_			
	Au–P		Au–Au		Au–P/S		Au–Au		Au–S		Au–Au	
	CN	*R* (Å)	CN	*R* (Å)	CN	*R* (Å)	CN	*R* (Å)	CN	*R* (Å)	CN	*R* (Å)
Pure clusters	2.6 (4)	2.34 (0)	2.3 (2)	2.81 (5)	1.2 (2)	2.29 (1)	2.0 (7)	2.79 (2)	1.1 (2)	2.31 (1)	0.8 (3)	2.78 (5)
Fresh	1.9 (4)	2.30 (2)	3.2 (6)	2.81 (1)	0.8 (1)	2.31 (1)	4.3 (5)	2.82 (8)	1.2 (2)	2.30 (0)	3.3 (5)	2.83 (1)
COox150 °C	0.6 (3)	2.27 (4)	9.9 (6)	2.85 (5)	0.6 (2)	2.31 (3)	5.8 (9)	2.83 (1)	0.9 (2)	2.30 (1)	5.5 (6)	2.84 (1)
COox200 °C	0.2 (5)		9.8 (9)	2.84 (5)	0.0 (0)		11.2 (9)	2.85 (5)	0.1 (1)		10.4 (6)	2.84 (0)
Pret250 °C	0.2 (3)		11.3 (6)	2.85 (5)	0.2 (1)		11.0 (6)	2.85 (0)	0.1 (1)		10.2 (6)	2.85 (0)
COox250 °C	0.1 (2)		10.6 (5)	2.85 (5)	0.1 (1)		10.8 (6)	2.85 (0)	0.1 (3)		10.6 (11)	2.85 (1)
COox250 °C rep	0.3 (2)		10.9 (9)	2.85 (5)	0.1 (1)		11.0 (10)	2.85 (5)	0.1 (2)		10.1 (10)	2.84 (1)
COox300 °C	0.2 (2)		8.9 (5)	2.84 (5)	0.2 (1)		10.3 (10)	2.85 (5)	0.1 (2)		10.3 (12)	2.84 (1)

The X-ray absorption near edge structure spectra (XANES) are shown in Fig. S1 (ESI†). The unsupported Au nanoclusters (dotted black line) clearly showed peaks corresponding to Au–S/P and Au–Au distances. For the unsupported Au_11_ clusters, the estimated Au–Au and Au–P bond lengths were within the same region as those of similarly sized phosphine protected Au nanoclusters.^12,28,29^ Moreover, while a Au–Au coordination number (CN) of 2.3 seems quite reasonable for a cluster of that size,^28^ the Au–P CN was with 2.6 much higher than the expected one of 0.63–0.73. The origin of this difference could not be resolved, however, a comparable overestimation was also observed for the CN_Au–P/S_ in Biico Au_25_/CeO_2_. Au_11_/CeO_2_ exhibited a decrease in the CN_Au–P_ and a slight increase in the CN_Au–Au_, indicating signs of ligand loss already during the supporting process. After pretreatment and reaction at 150 °C, the *R*-space and XANES spectra changed significantly (Fig. 2 and Fig. S1, ESI†), consistent with previous work showing that partial removal of ligands hinders the stabilization of the cluster.^30,31^ This suggests further ligand loss and increasing size of the remaining bare Au core during the reaction. No significant differences are observed at 200 °C with respect to 150 °C, while increasing to 250 °C leads to an increase in CN_Au–Au_. In this case, there are no representative changes between pretreatment, reaction and three reaction cycles (rep), indicating the stabilization of the clusters. The increase in bond distance *R*_Au–Au_ from 2.81 to 2.85 Å can be related to the cluster growth, as already observed in previous work.^4,5,30^ However, the CN_Au–Au_ is still smaller than fcc gold (CN ≅ 12), and so are the bond lengths (*R* > 2.9).^32^

For the mixed ligand shell cluster Biico Au_25_, the average CNs for Au–P/S and Au–Au were found to be 1.2 and 2.0, respectively. This presents a deviation from published values for similar icosahedral core structures.^20,33^ However, it has been reported previously that the fitted CN_Au−Au_ values of ligand-protected clusters may be too small owing to differences in the bond lengths within the cluster structure.^34–39^ The average bond lengths of 2.3 Å (Au–P/S) and 2.8 (Au–Au) are nevertheless in good agreement with literature.^20^ Similar to Au_11_/CeO_2_, a decrease of the CN_Au−P/S_ and an increase of the CN_Au−Au_ were noticed upon supporting, indicating a first structural change, presumably initiated by a first detachment of ligands.

This trend then further continued with rising temperatures; however, Biico Au_25_/CeO_2_ clearly exhibited higher thermal stability than Au_11_/CeO_2_: in this case, the major increase in the Au–Au CN appeared at 200 °C instead of 150 °C. Afterwards, the structure of the Au core did not seem to change significantly besides a slight reduction in the CN_Au–Au_ at 300 °C.

Finally, the evolution of the Au_25_/CeO_2_ catalysts also follows a quite similar trend. The unsupported cluster has a fitted CN_Au−S_ of 1.1 and a CN_Au−Au_ of 0.8. While the former is in agreement with literature^20^ – as are both bond lengths^20,35^ – again a significant underestimation is found for the CN_Au−Au_. Upon immobilizing the clusters on the ceria support, only marginal alterations were observed for the Au–S bonds, indicating that the ligand sphere should be mostly intact at this point. However, at the same time, the CN_Au−Au_ increased to 3.3 and the bond lengths to 2.83, suggesting structural changes due to contact with CeO_2_. The on-set of ligand desorption was noticed at 150 °C through a decreasing CN_Au−S_ and an increasing CN_Au−Au_ and seemed completed at 200 °C.

This general trend of ligand removal and subsequent slight growth of Au clusters during oxidative pretreatment and reaction has already been observed using EXAFS by several groups.^5,8,10,12,19,20,31^ This same trend can also be observed in the XANES spectra of all three catalysts (see Fig. S1, ESI†). For the unsupported clusters, a more intense white line feature (≈11924 eV) was detected for the more positively charged P-protected clusters.^12,20^ Upon supporting and thermal oxidative treatment, the white line feature was reduced, indicating the Au core becoming more metallic.^12^ For all cluster catalysts, the spectrum converged toward the one metallic gold, denoting the removal of partially charged gold related to the Au–ligand bonds, as well as due to an increase in cluster size. It should be noted that the CN values for Au–Au after reaction at 250 °C suggest that the average particles sizes were somewhat larger^20,40^ than the 2–3 nm estimated by (S)TEM for Biico Au_25_/CeO_2_ and Au_25_/CeO_2_ and more toward the 4–7 nm estimated for Au_11_/CeO_2_.^7^ However, the average bond lengths of 2.84–2.85 Å are in good agreement with the 2–3 nm estimated from microscopy.^32^ Moreover, both the CN_Au−Au_ and the Au–Au bond lengths of all three samples after reaction at 250 °C showed very similar values, which indicates that they are reasonably close in size.

Based on XAS, it seems that the process of ligand detachment from Au is completed at 200 °C (COox200 °C) for all three clusters. None of the samples showed significant Au–ligand contributions after oxidative pretreatment at 250 °C (pret250 °C) either. In good agreement, CO adsorbed on Au was observed by *in situ* transmission IR spectroscopy for all samples after pretreatment at 250 °C, which indicated that accessible, unprotected Au surface was available at this point.^7^ It thus seems likely that the ligands migrated to the ceria surface already at temperatures below 200 °C, but were not completely decomposed to CO_2_ and subsequently removed from the system unless higher temperatures were applied, as evidenced by our previous experiments.^7^ It also further affirms that their presence on the support, most likely adjacent to the Au clusters, is a crucial factor for the catalytic activity of these cluster catalysts in CO oxidation, since no striking difference in the Au–Au bond lengths or coordination numbers was observed among the investigated catalysts after reaction at 250 °C. Similar migration of either phosphine^12,21,41^ or thiolate^5,31^ ligands has also been reported for other systems before.

Interestingly, after reaction at 300 °C, the CN_Au−Au_ values for Au_11_/CeO_2_ and Biico Au_25_/CeO_2_ (slightly) decreased with respect to the values obtained after 250 °C, even after reaction cycles (rep), while it remained almost constant for Au_25_/CeO_2_ (within error range). The rather pronounced decrease for the Au_11_/CeO_2_ samples is also corroborated by changes in the XANES region (Fig. S1, ESI†), which shows an increase in the white line feature. This points toward the formation of more oxidized (smaller) particles.

Previous works have noted that the detachment of the ligand motifs (–S–Au–S–Au–S–) lead to SO_*x*_^5,31^ species and single or small Au clusters near the main Au cluster core. Therefore, one hypothesis could be that the decrease in the CN_Au−Au_ observed at 300 °C could be related to the formation of small Au clusters stabilized on the surface around the main cluster core structure once the temperature rises to 300 °C. Previous work on Au_38_ clusters supported on CeO_2_ has found that this effect seemed to be mediated by the sulphur residues, which appeared to migrate from the cluster surface to the support at pretreatment temperatures of 250 °C, drawing parts of the Au atoms with them.^5^ It is possible that a similar effect might result in the formation of smaller particles also in the case of the (partially) phosphine-protected clusters.

It is worth noting that this decrease in CN_Au–Au_ appears at the same temperature at which the main ligand decomposition/removal process seems to take place according to our previous study.^7^ It thus might be possible that the P- and S-ligand residues could contribute to the formation of smaller gold particles. This would also explain the apparent changes in the Au_11_/CeO_2_ XANES spectrum at 300 °C. Considering that these spectroscopic techniques represent the average signal, the differences in the values could be explained. This change also correlates with a strong increase in catalytic CO oxidation activity of both Au_11_/CeO_2_ and Biico Au_25_/CeO_2_ (Fig. 1).

**Fig. 3:**
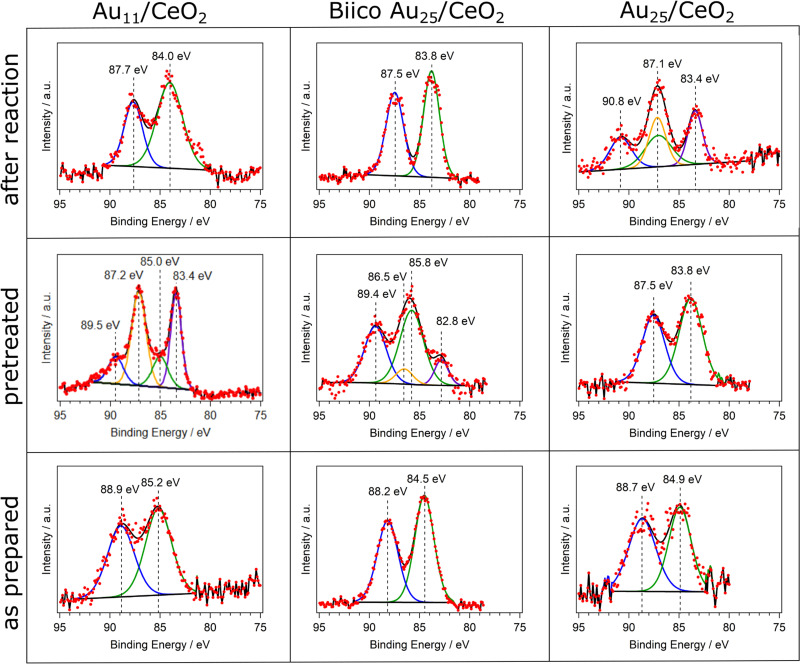
Overview over the XPS measurements of the Au 4f region of the clusters. Each cluster was analyzed as prepared (bottom row), after oxidative pretreatment (middle row) and after CO oxidation experiments at 250 °C (top row).

In order to complete the study on the oxidation state after pretreatment and reaction at 250 °C, XPS analysis was performed and is displayed in Fig. 3. Au_11_/CeO_2_ shows a binding energy of 85.2 eV for the Au 4f_7/2_ transition, close to reported values in literature^21,42^ with the same cluster but using different support. However, it is significantly larger than the binding energy of bulk Au with around 84.0 eV. It is known that the binding energies of Au in such small clusters can increase due to initial and final state effects.^43^ These binding energy values suggest charged Au that can be related to the Au atoms bonded to the ligands. Upon pretreatment and removal of the ligands, a higher proportion of metallic Au is denoted by the decrease in the binding energy value from 85.2 eV to 83.4 eV for the 4f_7/2_ transition. However, a second component related to charged gold is still present in the spectrum (Au 4f_7/2_ transition at 85.0 eV), which could be denoted to Au atoms bonded to P or strongly interacting with the CeO_2_ surface. This is in agreement with reported studies by XPS with similar systems.^21^ Furthermore, it also connects to our previous results with CO adsorption experiments follow by *in situ* IR, showing two different Au species after pretreatment.^7^ The comparison of the respective XPS peak areas showed that around 68% of the Au species were in metallic state. After the reaction, only one component was present with a binding energy of 84.0 eV. Due to the relatively low binding energy, the Au species appeared to be in metallic state, in agreement with the XAFS data.

In the case of BiicoAu_25_/CeO_2_ after supporting (as prepared), there is good agreement with literature values for the unsupported clusters,^26^ indicating that the structure is largely preserved at this step, which can also be seen in the EXAFS and XANES spectra (Fig. 2 and Fig. S1, ESI†). After the oxidative pretreatment, the binding energy of the 4f_7/2_ transition increased from 84.5 eV to 85.8 eV. Similar to Au_11_/CeO_2_, a second component is present after oxidative pretreatment (Fig. 3), which has a rather low 4f_7/2_ binding energy of 82.8 eV. This most likely metallic component made up about 17% of the analyzed gold. After CO oxidation, the binding energy decreased to 83.8 eV due to the Au core becoming more metallic during the reaction.

For Au_25_/CeO_2_, a similar trend as with Au_11_/CeO_2_ can be observed, though not as extreme. The initial binding energy of the Au 4f_7/2_ peak is 84.9 eV, which is slightly higher compared to the literature.^17,30^ The oxidative pretreatment leads to a decrease in binding energy, indicating formation of metallic gold, presumably due to ligand removal. This has previously also been observed for similar catalyst systems and pretreatments.^5,17^ After the reaction, the Au is present in two forms. A metallic component with a binding energy of 83.4 eV for the Au 4f_7/2_ transition and an oxidic component with a binding energy of 87.1 eV for the 4f_7/2_ transition. It should be noted that the peak for this transition is overlapping with the 4f_5/2_ peak for the metallic component, hence only three peaks are present in the spectrum. The metallic component makes up around 45% of the Au present in the sample. However, due to the peaks overlapping this value is not very precise. Furthermore, the still oxidic component is shifted to an extraordinary high binding energy indicating a very electronegative binding partner. However, *in situ* IR studies of CO adsorption on the same catalyst did not find any presence of Au^+^ species,^7^ and, similarly, XANES data also did not reveal Au^+^. This discrepancy might thus be due to the poorer data quality in this measurement (in 1 mbar N_2_ instead of UHV).

Summarizing, a general trend toward more metallic gold species is observed in the XPS measurements for all catalysts. For Au_11_/CeO_2_ and Biico Au_25_/CeO_2_, oxidized species can still be detected after oxidative pretreatment. This could indicate that their ligand removal process proceeds differently to the one of the fully thiolate-protected cluster. After reaction, a shift toward lower binding energies has occurred for all clusters (aside from the oxidized component in the Au_25_/CeO_2_ spectrum). This is in good agreement with the process of ligand removal and consequently formation of slightly sintered Au particles.

## Conclusions

The state of the Au core of Au_11_, Biico Au_25_ and Au_25_ clusters on ceria used as catalysts for CO oxidation was probed at different stages of the process by *ex situ* EXAFS and XPS. While previously a strong activity difference at 250 °C was noticed,^7^ the clusters showed similar Au–Au coordination numbers and bond lengths after reaction at this temperature. Moreover, most of the Au surface appeared to be free of ligands and thus accessible already from 200 °C onwards for all clusters. Furthermore, significant changes in the Au oxidation state were noticed after oxidative pretreatment at 250 °C by XPS, which shows that the treatment indeed affected the Au core (*i.e.* the ligand shell is not intact anymore at this point). After reaction, formation of mostly metallic gold was observed for all samples. This affirms the previous assumption that blocking of active sites by ligand residues might be an important factor for the difference in catalytic activity at 250 °C.^7^ After reaction at 300 °C, a decrease in the CN_Au–Au_ was noted for both Au_11_/CeO_2_ and Biico Au_25_/CeO_2_ by EXAFS, which could be related to formation of smaller particles. This structural change appears at the same temperature for which an significant increase in CO oxidation activity was noticed, as well as for which the main removal of the ligand framework from the catalytic system takes place.^7^ Therefore, a synergistic effect between the nature of the support, the cluster structure, and the ligand shell seems to determine the surface configuration of the gold nanocluster catalysts after pretreatment and during the reaction, which in turn determines the catalytic behaviour. These results emphasize once again the complexity of the structural changes of such supported nanoclusters during pretreatment and catalytic reaction, and show that they are driven by different effects. A combination of different (*in situ*/*operando*) techniques is therefore crucial to understand the evolution of the system.

## Author contributions

Conceptualisation – V. T., N. B., G. A., methodology – N. B., V. T., F. S., C. R., C. M., investigation – N. B., F. S., M. S. S., M. P., G. A., formal analysis – N. B., F. S., C. M., G. A., writing (original draft) – V. T., F. S., N. B., C. R., G. A., C. M., writing (review & editing) all, resources – N. B., C. R., M. S. S., G. A., supervision – N. B.

## Conflicts of interest

There are no conflicts to declare.

## Supplementary Material

CP-025-D2CP04498F-s001
