# Supplementary material for: Structural evolution after oxidative pretreatment and CO oxidation of Au nanoclusters with different ligand shell composition: a view on the Au core
Source: Phys Chem Chem Phys. 2023 Jan 5;25(5):3622–8. doi: 10.1039/d2cp04498f (PMC9890638; doi:10.1039/d2cp04498f)
Supplement: CP-025-D2CP04498F-s001 [file CP-025-D2CP04498F-s001.pdf]

Electronic Supplementary Material (ESI) for Physical Chemistry Chemical Physics.
This journal is © the Owner Societies 2023

Electronic Supplementary Information

Structural evolution after oxidative pretreatment and CO oxidation of Au nanoclusters with different ligand shell composition: a view on the Au core

Vera Truttmann [θ,a,‡] Florian Schrenk [θ,b] Carlo Marini,[c] Mireia Palma,[d] Maricruz Sanchez-Sanchez,[d] Christoph Rameshan,[a,b] Giovanni Agostini,[c] Noelia Barrabés [a,*]

a. *Institute of Materials Chemistry, TU Wien, Getreidemarkt 9/165, 1060 Vienna, Austria.*

b. *Chair of Physical Chemistry, Montanuniversität Leoben, Franz-Josef-Straße 18, 8700 Leoben, Austria*

c. *ALBA Synchrotron Light Facility, Carrer de la Llum 2-26, 08290 Cerdanyola del Valles, Barcelona, Spain.*

d. *Institute of Chemical, Environmental and Bioscience Engineering, TU Wien, Getreidemarkt 9/166, 1060 Vienna, Austria*

‡ *Current address: Institute for Chemical Technology and Polymer Chemistry, Karlsruhe Institute of Technology, Engesserstraße 20, 76131 Karlsruhe, Germany.*

ϑ *Shared first co-authorship*

Correspondence: noelia.rabanal@tuwien.ac.at

1 XANES spectra and EXFAS fitting details

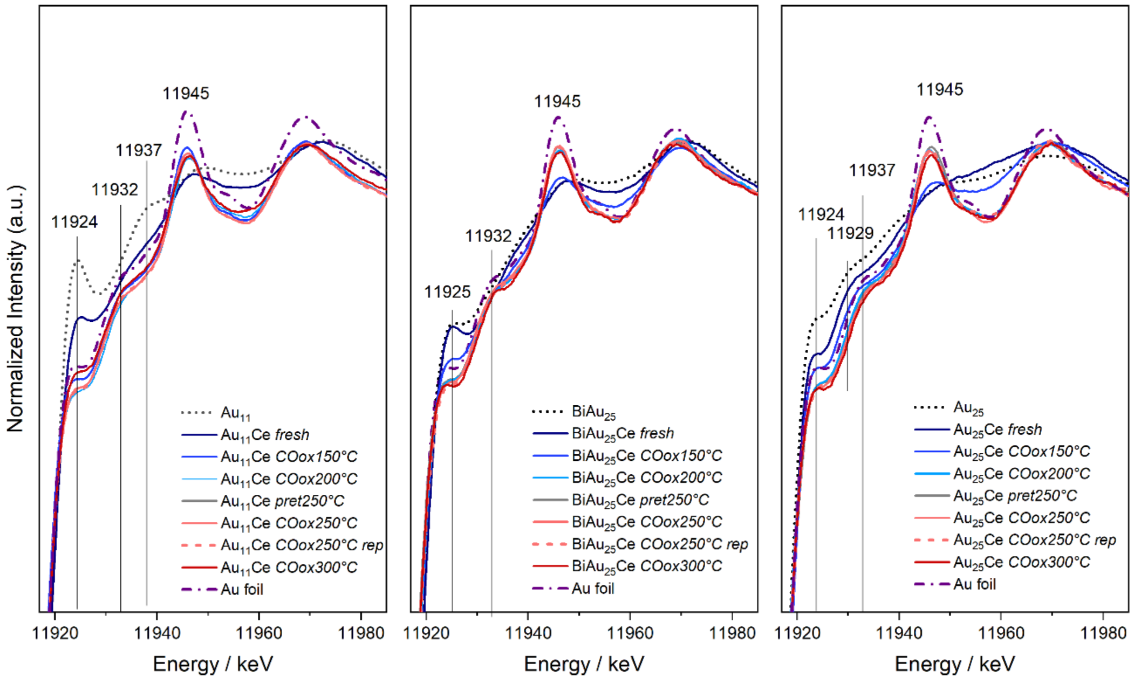

Figure S1: XANES spectra of the catalysts at Au L_3-edge, from left to right: Au_{11}/CeO_2, Biico Au_{25}/CeO_2 and Au_{25}/CeO_2. Each graph shows the spectrum of the unsupported clusters (black dotted), the supported clusters (dark blue), the clusters after pretreatment and reaction at different temperatures (blue and reddish solid lines), the catalyst after pretreatment at 250 °C but before reaction (grey), after 3 consecutive CO oxidation runs (red dashed) and the Au foil reference (violet dashed).

Table S1: Parameters for EXAFS fitting

amp	enot	σ^2(Au–P)	σ^2(Au–S)	σ^2(Au–Au)
0.89±0.22	2.64±0.25	0.013±0.002	0.005±0.001	0.009±0.000

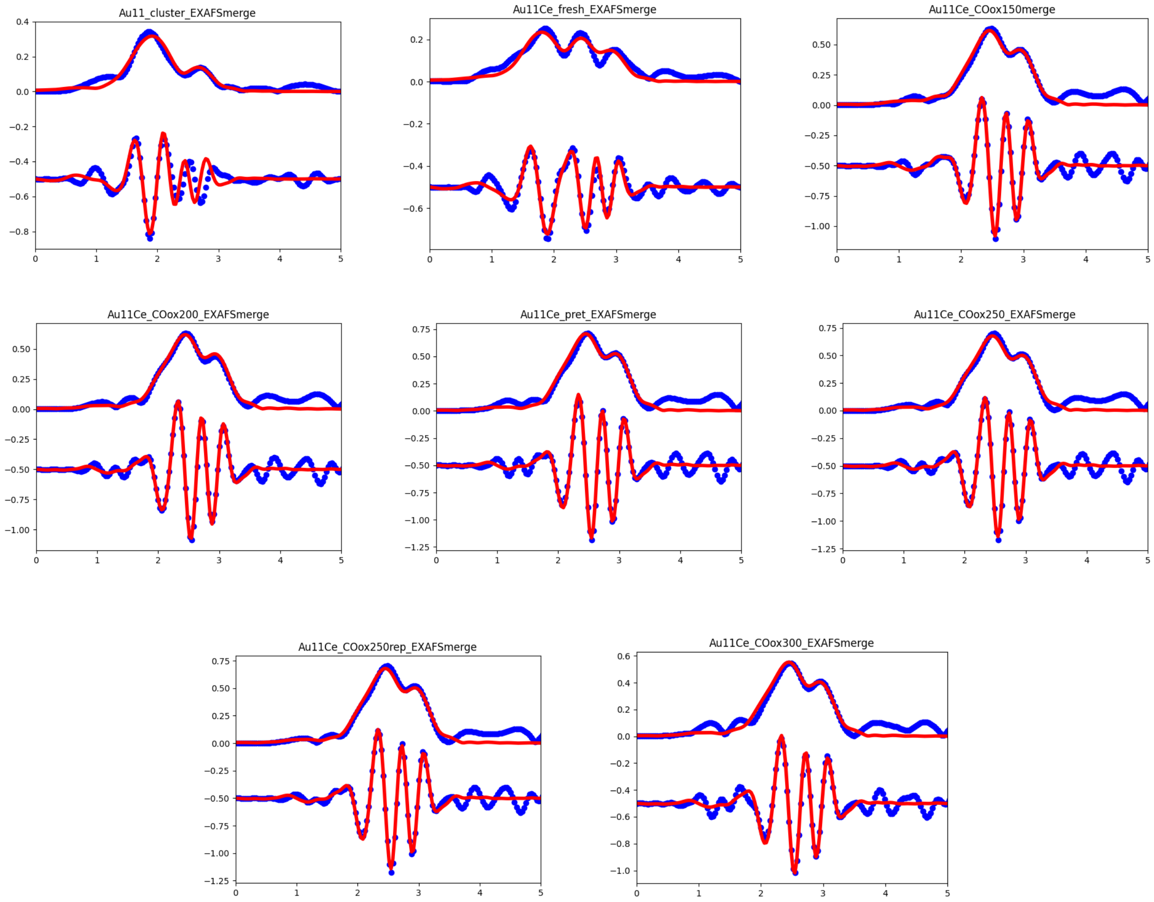

Figure S2: EXAFS fitting of Au_{11}/CeO_2 in R space: measured spectrum = blue, fit = red.

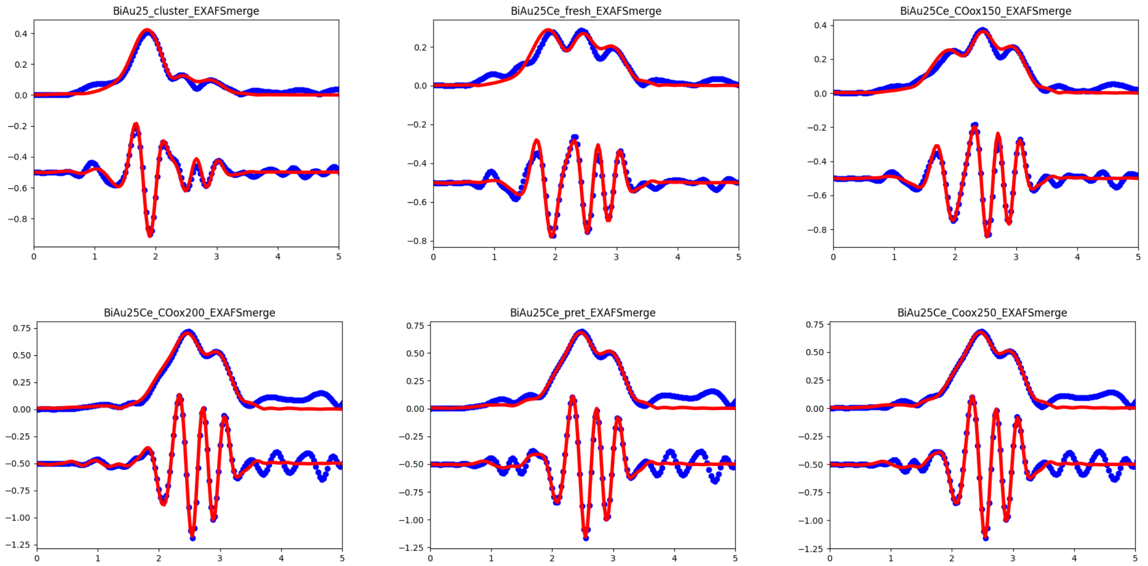

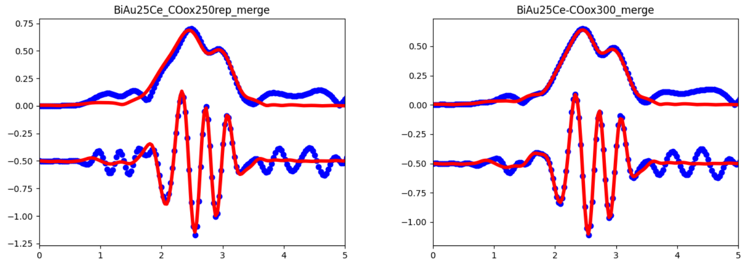

Figure S3: EXAFS fitting of Biico Au_{25}/CeO_2 in R space (Å): measured spectrum = blue, fit = red.

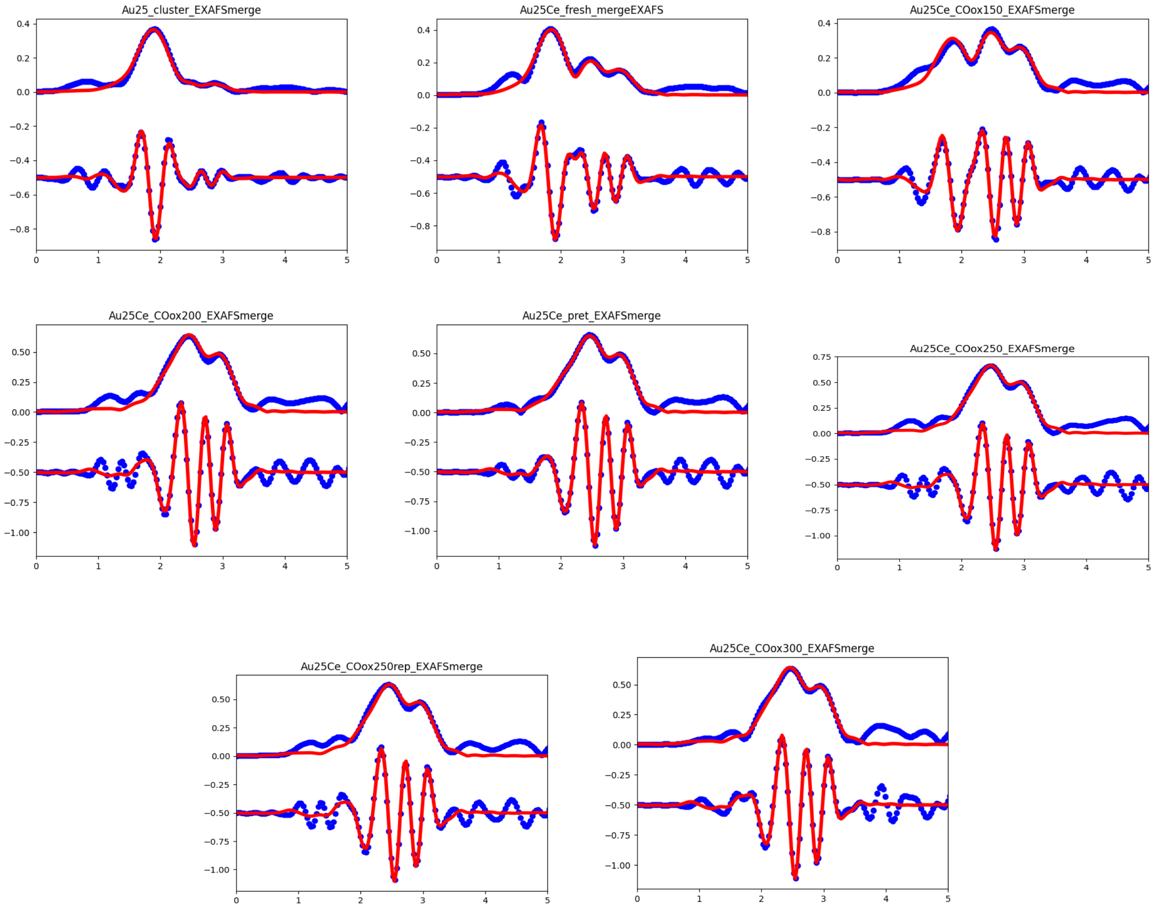

Figure S4: EXAFS fitting of Au_{25}/CeO_2 in R space: measured spectrum = blue, fit = red.

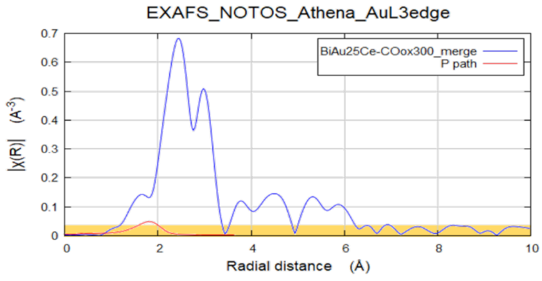

Figure S5: Demonstration of the noise level (yellow area) in the EXAFS fitting.

2 Additional XPS spectra

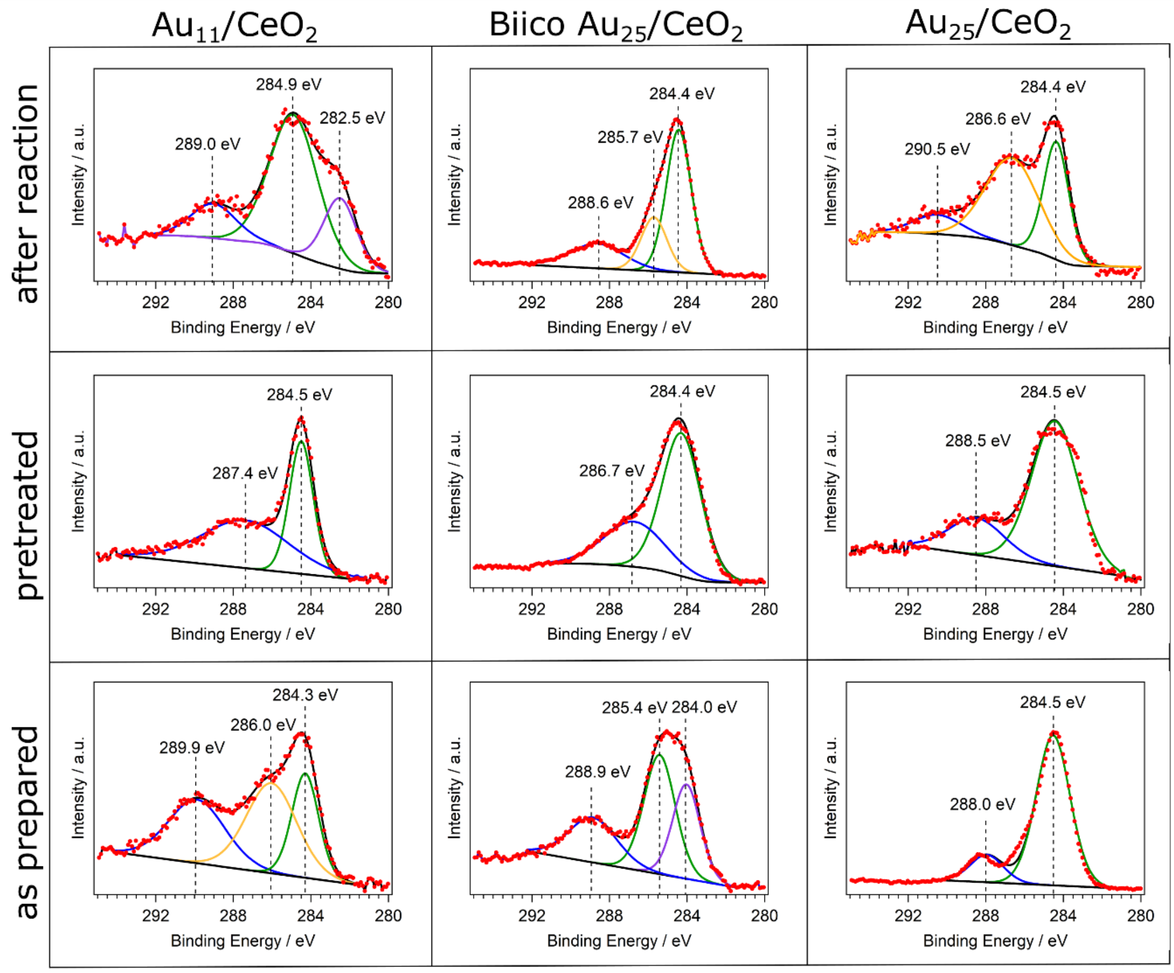

Figure S6: Overview over the XPS measurements of the C_{1s} region of the clusters. Each cluster was analyzed as prepared (bottom row), after oxidative pretreatment (middle row) and after CO oxidation experiments (top row). The different colors in the fitting indicate adventitious carbon (green) and other species with different binding energies (blue > orange > violet).

The analysis of the C 1s makes it evident that all samples exhibit adventitious carbon as they were exposed to air before the measurement. This component (marked in green in Figure S6) is ubiquitous and well-studied.[1] Several other components are visible in the spectra which were grouped into three groups of similar binding energy: the highest binding energy (290.5 eV to 287.4 eV) marked in blue, intermediate binding energy (around 286 eV) marked in orange and the lowest observable binding energy (284.0 eV to 282.5 eV) marked in violet. The component with the highest binding energy is present in all recorded spectra. It is most likely a carbon-oxygen compound sitting on the surface.[2] As it is not removed by the oxidative pretreatment and also present after the reaction a structure bond to the CeO_2 support is very likely. This is in agreement with previous transmission IR measurements of the same catalysts that indicated presence of different (hydro)carbonate and formate compounds.[3] The group with intermediate binding energy present in Au_{11}/CeO_2 after supporting and in Biico Au_{25}/CeO_2 and Au_{25}/CeO_2 after reaction also indicate formation of oxidized carbon species. This component can be related to residues from supporting (e.g. solvent or ligand residues) or CO oxidation. An exact assignment cannot be done as the C 1s spectra allows for a lot of interpretation at this binding energy.[4]

Table S2. Results of the XPS fitting results for the Au4f region of the cluster catalysts at different stages.

	Au_{11}/CeO_2				Biico Au_{25}/CeO_2				Au_{25}/CeO_2			
	Component 1		Component 2		Component 1		Component 2		Component 1		Component 2	
as prepared	88.9 eV	85.2 eV	-	-	88.2 eV	84.5 eV	-	-	88.7 eV	84.9 eV	-	-
pretreated	89.5 eV	85.0 eV	87.2 eV	83.4 eV	89.4 eV	85.8 eV	86.5 eV	82.8 eV	87.5 eV	83.8 eV	-	-
after reaction	87.7 eV	84.0 eV	-	-	87.5 eV	83.8 eV	-	-	90.8 eV	87.1 eV	87.1 eV	83.4 eV

Table S3. Results of the XPS fitting results for the C1s region of the cluster catalysts at different stages.

	Au_{11}/CeO_2				Biico Au_{25}/CeO_2				Au_{25}/CeO_2			
	C1	C2	C3	C4	C1	C2	C3	C4	C1	C2	C3	C4
as prepared	284.3 eV	289.9 eV	286.0 eV	-	284.5 eV	288.9 eV	-	284.0 eV	284.5 eV	288.0 eV	-	-
pretreated	284.5 eV	287.4 eV	-	-	284.4 eV	286.7 eV	-	-	284.5 eV	288.5 eV	-	-
after reaction	284.9 eV	289.0 eV	-	282.5 eV	284.4 eV	288.6 eV	285.7 eV	-	284.5 eV	288.0 eV	-	-

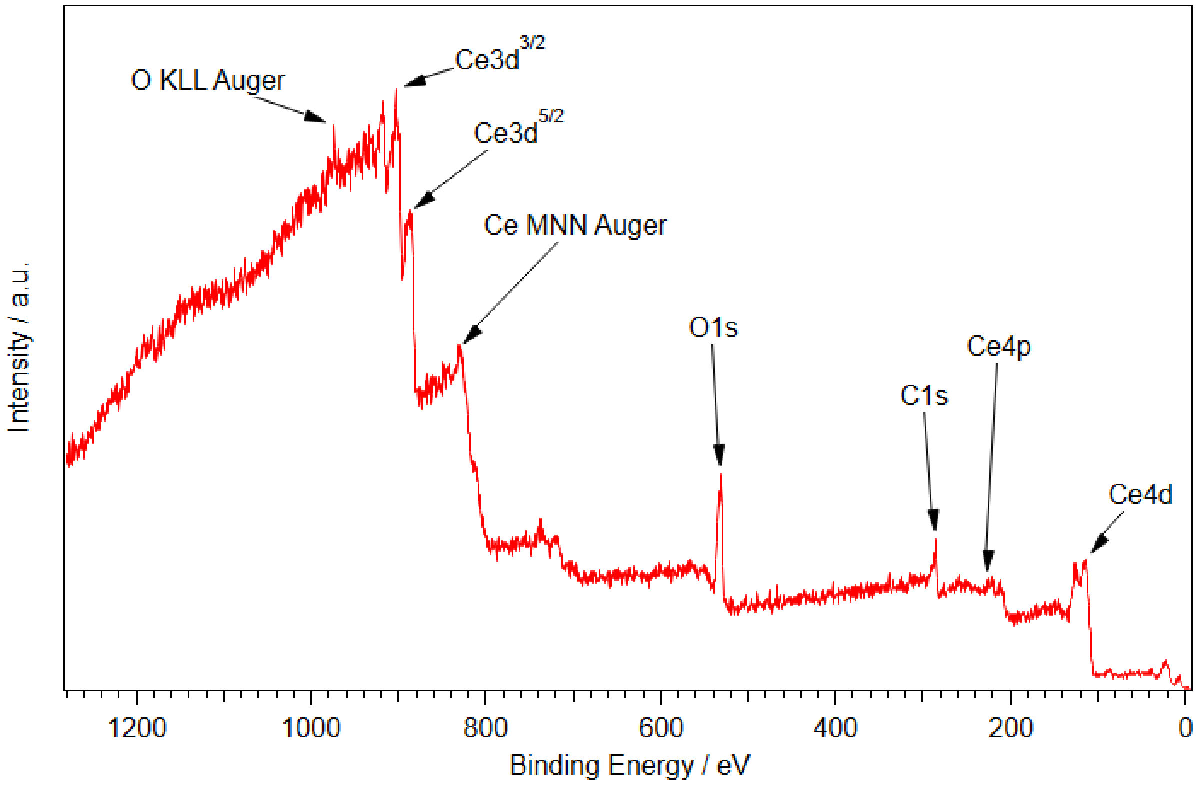

Figure S7: Exemplary XPS survey scan of Au clusters supported on CeO_2. Due to the low loading (wt%), only peaks related to ceria and carbon can be observed. No impurities are present in significant amount.

References

1. T. L. Barr and S. Seal, *Journal of Vacuum Science & Technology A*, 1995, **13**, 1239-1246.
2. A. R. Gonzalez-Elipe, J. P. Espinos, A. Fernandez and G. Munuera, *Applied Surface Science*, 1990, **45**, 103-108.
3. V. Truttmann, H. Drexler, M. Stöger-Pollach, T. Kawawaki, Y. Negishi, N. Barrabés and G. Rupprechter, *ChemCatChem*, 2022, **14**, e202200322.
4. X. Chen, X. Wang and D. Fang, *Fullerenes, Nanotubes and Carbon Nanostructures*, 2020, **28**, 1048-1058.
